# Asymmetric effects of crude oil prices and USD exchange rate on precious metals returns：Evidence from pre and during COVID-19 outbreak

**DOI:** 10.1016/j.heliyon.2023.e21996

**Published:** 2023-11-02

**Authors:** Yilin Wang, Jinyu Chen, Xiaohang Ren

**Affiliations:** a School of Business, Central South University, Changsha, 410083, China; b Institute of Metal Resources Strategy, Central South University, Changsha, 410083, China

**Keywords:** Precious metals returns, Oil price, USD exchange rate, Asymmetric effects

## Abstract

Trading in commodities such as precious metals and crude oil is vital to the economy. Frequent exchange rate fluctuations have led to constant changes in commodity prices since 2000. Using quantile regression, this paper examines the impact of oil prices and the US dollar exchange rate on gold, silver, platinum, and palladium from January 1, 2013 to 5 May 2023. Oil prices positively affect precious metals returns, and positive and negative oil price shocks are asymmetric. Exchange rate movements negatively affect precious metal returns. In addition, gaps in the existing literature are filled by analyzing the effects of oil prices and the exchange rate on precious metals before and during COVID-19. This paper provides substantial evidence for revising the impact of the crisis.

## 1 Introduction

Oil and precious metals are considered globally influential investment assets and commodities [1]. The flare of their price will inevitably affect the world energy market and economic development [2]. Since precious metals and crude oil are products priced in the US dollar, the USD exchange rate could also push oil and precious metals returns [3]. For example, when oil prices rise, it will cause inflation and depreciation of the dollar. In this way, looming inflation will push up precious metals prices because many investors see them as a safe haven for money, and they can possess value in times of inflation [4,5]. In this context, precious metals, crude oil, and exchange rates form interconnected financial markets [6,7]. Considering the rapid financialization of commodities and market integration, it will be informative to examine the overall influence of oil prices and the USD exchange rate on the precious metals returns in a diverse context [7].

In 2020 the world experienced a substantial public health crisis, the COVID-19 epidemic. During this period, an oil price earthquake occurred in the Middle East, and the crude oil price plummeted. For example, WTI crude was down 30 %, Brent 28.32 % on the day, and oil-related stocks such as EOG Resources fell 50 % [8]. The demand for commodities fell sharply in the first quarter of 2020, and investors in commodity markets felt more anxious about the future prices of commodities [[9], [10], [11]]. Given that the COVID-19 epidemic is the source of systemic risks and is highly destructive, it is necessary to re-examine the impact on precious metals in a crisis state to study the financial impact of COVID-19 further.

Taking the dynamics of the precious metals, oil prices, and USD exchange rate into account, the primary studies have focused on them. Firstly, by studying the association between oil prices and precious metals (especially gold), previous studies have revealed that the interaction is nonlinear and asymmetric [[12], [13], [14], [15]]. Specifically, oil and gold share similar price trends and could be used to predict prices mutually [10,15,16]. In addition, by dividing the oil price into rise and fall to further identify the asymmetry, studies pointed out that the gold price was more subtle than the positive oil price [17,18]. Uddin et al. [19] decomposed oil prices into three channels and discovered the significant positive effects of supply and demand shocks and the adverse effects of risk shocks on precious metals returns. Ulteriorly, Baruník et al. [20] used several critical events and found they could cause structural disruption between oil and precious metal prices. The dynamic correlation between the two assets helped design policies that avoid the risk of pass-through during economic uncertainty [21]. However, some literature didn't show a significant relationship between precious metals and oil prices [22,23], and this may be due to the research interval because Sari et al. [1] figured that the precious metals responded significantly positively to oil prices in the short term, the impact would dissipate in the long run.

Secondly, continued joint movements in commodity prices and exchange rates are of great interest to investors; therefore, existing studies have examined the correlation between the USD exchange rate and precious metals. Batten et al. [24] found that currency factors caused the gold price fluctuations, and these results were crucial for subverting foreign exchange returns, especially during recessions, as commodities were alternatives to the dollar [[25], [26], [27], [28]]. Mainly, Beckmann et al. [29] and He et al. [30] figured out the frequent fluctuations in the exchange rate, and the results showed that the hedging function of gold made the price rise. Nevertheless, this can be explained by inflation and a solid mutual causality exists between inflation and exchange rates [[31], [32], [33]].

Last but not least, many scholars have begun to focus on the affiliation of precious metals, crude oil prices, and exchange rates by applying different econometric models. For example, some scholars adopt a wavelet approach named ARDL to discover the cointegration among these three series [1,7,34]. Applying these econometric models, Wang and Chueh [35] and Jain and Biswal [36] pointed out that the decline in international oil and gold prices will cause the ruble to depreciate. Moreover, Bedoui et al. [37] proposed that the dependence between gold, oil, and the exchange rate is more substantial during the crisis period.

There are some limitations in the existing literature. On the one hand, limited literature includes oil in the commodity market and exchange rates in financial markets in one framework to consider their effects on precious metals. The effects of oil are deeply researched [[38], [39], [40]]. However, some studies neglect to consider financial market indicators. The trend towards the financialization of commodities has become more pronounced in recent years, and the impact of financial indicators such as currencies on precious metals should be studied more extensively [3]. On the other hand, many methods are used in the existing literature, such as GARCH and spillover models, which consider the market as one state. The commodity markets are dynamic, constantly rising, stable, and falling in different market conditions [41]. Switching between states, the linear model alone can't describe the differences in the impact of different market states [40]. In summary, this paper builds on existing research by incorporating energy, commodities, and currency into the same framework and using quantile regression to explore the response of precious metals in different market states to oil and exchange rate.

This research is mainly aimed at solving the following problems: 1) Do changes in oil prices and USD exchange rate impact precious metals futures returns, and how? 2) Has the shock of COVID-19 changed the relationship? A quantile regression (QR) model is adopted to inspect the asymmetric effects of crude oil prices (OP) and the USD exchange rate (ER) on precious metals returns (PMR) pre- and during the COVID-19 outbreak. This paper contributes in three significant ways. First, this paper provides a comprehensive study to theoretically enrich the energy-commodity and commodity-currency nexus research. The heterogeneity in reactions between PMR to OP and ER is fully considered. Second, the quantile regression (QR), examines the non-linear impact under different market conditions: bearish, normal, and bullish is examined. Finally, this paper investigates the heterogeneous effects of the worldwide crisis COVID-19 from 2020 to 2023 by comparing the impact before and during the COVID-19 epidemic.

## 2 Methodology

### 2.1 Standard OLS models

This paper investigates whether and how OP and ER affect precious metal returns. Firstly, presenting the standard OLS models, equation (1) (2) are putting forward.

(1) $$R_{i,t}=\alpha_{i}+\beta_{1}{WTI}_{t}+\beta_{2}{FFR}_{t}+\beta_{3}{VIX}_{t}+\varepsilon_{i,t}\text{,}$$

(2) $$R_{i,t}=\alpha_{i}+\beta_{4}{ER}_{t}+\beta_{2}{FFR}_{t}+\beta_{3}{VIX}_{t}+\varepsilon_{i,t}\text{,}$$

where $R_{i,t}$ represents the i th precious metals' return at time t. ${WTI}_{t}$ and ${ER}_{t}$ denote the price shocks of crude oil and exchange rate at time t. ${FFR}_{t}$ and ${VIX}_{t}$ stand for the interest rate and market instability index, respectively. $\beta_{1}$ and $\beta_{4}$ are the estimated parameters of the independent variables, while $\beta_{2}$ and $\beta_{3}$ are the estimated coefficients of the control variables. Considering the mutual influence of oil price and exchange rate [33,42,43], this paper regresses them separately. $\alpha_{i}$ is the constant term and $\varepsilon_{i,t}$ is the random error term.

In this paper, Equation (1) is improved by Equation (3) to explore the asymmetric impact of OP. Equation (1) assumes that OP have a symmetric impact on PMR, whereas previous literature reveals that a rise or fall in OP can affect commodities differently [44,45].

(3) $$R_{i,t}=\alpha_{i}+\beta_{1,1}{WTIZ}_{t}+\beta_{1,2}{WTIF}_{t}+\beta_{2}{FFR}_{t}+\beta_{3}{VIX}_{t}+\varepsilon_{i,t}\text{,}$$

where ${WTIZ}_{t}$ and ${WTIF}_{t}$ denote the increase and decrease of OP. According to Ishaan et al. [45] and Chen et al. [10], they can be defined as: ${WTIZ}_{t}=\max\left(0,{WTI}_{t}\right),{WTIF}_{t}=\min\left(0,{WTI}_{t}\right)$.

### 2.2 Quantile regression

The regression coefficients estimated by the standard OLS method can reveal how the dependent variable changes when the independent variable changes. However, the OLS method only gives conditional means of model parameters and cannot provide a complete description of the conditionally distributed dependent variable. Several studies have found asymmetric shocks to precious metals from oil prices and exchange rate [14,32,40], which implies that their relation cannot be discussed purely under a specific condition but should be considered dynamically. To overcome the flaws, this paper employs quantile regression (QR), according to Koenker and Bassett [46], Xiao et al. [47], and Das and Kannadhasan [44]. This model is widely applied because it can provide a more detailed view of variables and the asymmetric effects of conditions distributed at different quantiles [45,48,49].

According to Zhu et al. [50], Mokni [51] and Nusair and Olson [52], the basic quantile model is as follows:

(4) $$Qy_{\tau }\left(\tau 丨x_{i}\right)={x_{i}}^{\prime }\beta_{\tau }$$

Where 0< τ <1, $Qy_{\tau }\left(\tau 丨x_{i}\right)$ represents the conditional volatilities of the function, $x_{i}$ denotes the explanatory variables, $\beta_{\tau }$ is the parameters of the formula, which can be described as follows:

(5) $${\hat{\beta }}_{N}\left(\tau \right)=\mathrm{argmin}\beta_{\left(\tau \right)}\left(\sum_{i=1}^{N}\rho_{\tau }\left(y_{i}-x_{i}^{\prime }\beta \left(\tau \right)\right)\right)$$

Through the previous analysis, to study the impact of OP and ER on PMR in different market conditions and the asymmetric effect of rising and falling oil prices, the following quantile models are constructed based on Equation (1) (2) (3).

(6) $$Q_{R_{i,t}}\left(\tau 丨\alpha_{i},x_{it}\right)=\alpha_{i}+\beta_{1\tau }{WTI}_{t}+\beta_{2\tau }{FFR}_{t}+\beta_{3\tau }{VIX}_{t}+\varepsilon_{i,t}\text{,}$$

(7) $$Q_{R_{i,t}}\left(\tau 丨\alpha_{i},x_{it}\right)=\alpha_{i}+\beta_{4\tau }{ER}_{t}+\beta_{2\tau }{FFR}_{t}+\beta_{3\tau }{VIX}_{t}+\varepsilon_{i,t}\text{,}$$

(8) $$Q_{R_{i,t}}\left(\tau 丨\alpha_{i},x_{it}\right)=\alpha_{i}+\beta_{1,1\tau }{WTIZ}_{t}+\beta_{1,2\tau }{WTIF}_{t}+\beta_{2\tau }{FFR}_{t}+\beta_{3\tau }{VIX}_{t}+\varepsilon_{i,t}\text{.}$$

The quantiles could be divided into low (0.1, 0.2, 0.3), intermediate (0.4, 0.5, 0.6), and high quantiles (0.7, 0.8, 0.9) [50,53]. In this way, the quantiles respect three kinds of commodity market conditions: bearish, normal, and bullish.

## 3 Data and descriptive statistics

### 3.1 Dependent variables

Dedicated to studying the factors affecting the returns of precious metals, four kinds of precious metals are considered dependent variables, including gold, silver, platinum, and palladium. According to Hau et al. [54], the returns of precious metals can be defined as $R_{i,t}=\ln\;P_{i,t}-\ln\;P_{i,t-1}$. Where $P_{i,t}$ represents the price of precious metal i at time t. The data is collected at the New York Commodity Exchange (COMEX) [55].

### 3.2 Independent variables

Independent variables include oil price shocks and exchange rate. Since the West Texas Intermediate (WTI) market is one of the most widely used series, the WTI future price is the proxy variable of world oil prices, similar to You et al. [53]. Following Bagheri and Ebrahimi [56] and Andreasson et al. [57], this paper applies the U.S. dollar index to represent the USD exchange rate.

### 3.3 Control variables

Numerous studies have revealed the impact of interest rates and market volatility on PMR [[57], [58], [59], [60]]. To control for the effects of these two variables, this paper employs the Chicago Board Options Exchange Volatility Index (VIX) and the Federal Funds rate (FFR) as the proxy variables of market instability and interest rates.

### 3.4 Descriptive statistics

The sample period of this study spans January 1, 2013 to May 5, 2023, providing 2442 daily observations. The choice of research interval is based mainly on data availability. All the data series are sourced from the Wind database.

Table 1 summarizes the descriptive analysis of the data. The distinct kurtosis and skewness illustrate these sequences' sharp peaks and thick tails. Moreover, Jarque-Bera test results indicates that all sequences don't follow normal distribution. In this context, the standard OLS model may not be robust [53,60]. As we all know, quantile regressions are applied when the conditions required for linear regressions are violated (i.e., normality); descriptive statistics help us find the nonnormality of the data, which is conducive to quantile regression.

**Table 1:** Descriptive analysis.

	**Gold**	**Silver**	**Platinum**	**Palladium**	**WTI**	**ER**	**FFR**	**VIX**
**Mean**	0.00008	−0.00006	−0.00016	0.00033	0.00014	0.00011	0.00202	2.81926
**Maximum**	0.06255	0.07946	0.10107	0.20097	0.31963	0.02952	0.75000	4.41510
**Minimum**	−0.07124	−0.16279	−0.13336	−0.24196	−0.28180	−0.02089	−0.85000	2.21266
**Std. Dev.**	0.00974	0.01771	0.01615	0.02221	0.02881	0.00393	0.04670	0.33173
**Skewness**	−0.26961	−0.67492	−0.29471	−0.82781	0.27406	0.40455	5.12195	0.87071
**Kurtosis**	7.45444	10.68564	8.00614	15.58831	25.17359	9.03902	175.42930	4.06657
**Jarque-Bera**	2049***	6196***	2585***	16,403***	50,017***	3777***	3035895***	424***
**ADF test**	−48.635***	−49.439***	−32.691***	−45.466***	−49.334***	−56.749***	−52.091***	−6.611***
**PP test**	−48.629***	−49.441***	−48.823***	−45.693***	−49.343***	−56.829***	−52.024***	−6.595***
**Observations**	2442	2442	2442	2442	2442	2442	2442	2442

Note: *** represents 1 % level of significance.

The unit root test is applied to eliminate heteroscedasticity and to avoid false regression results. Among these, Dickey and Fuller (ADF) (1979), and Phillips and Perron (PP) (1988) tests are employed [61] (see Table 1). After testing the unit root, this paper employs the first difference logarithmic of Gold, Silver, Platinum, Palladium, ER, WTI, the logarithmic sequence of VIX, and the first difference of FFR.

## 4 Results and discussions

### 4.1 Effects of oil price shocks on precious metals for full sample

**Fig. 1:**
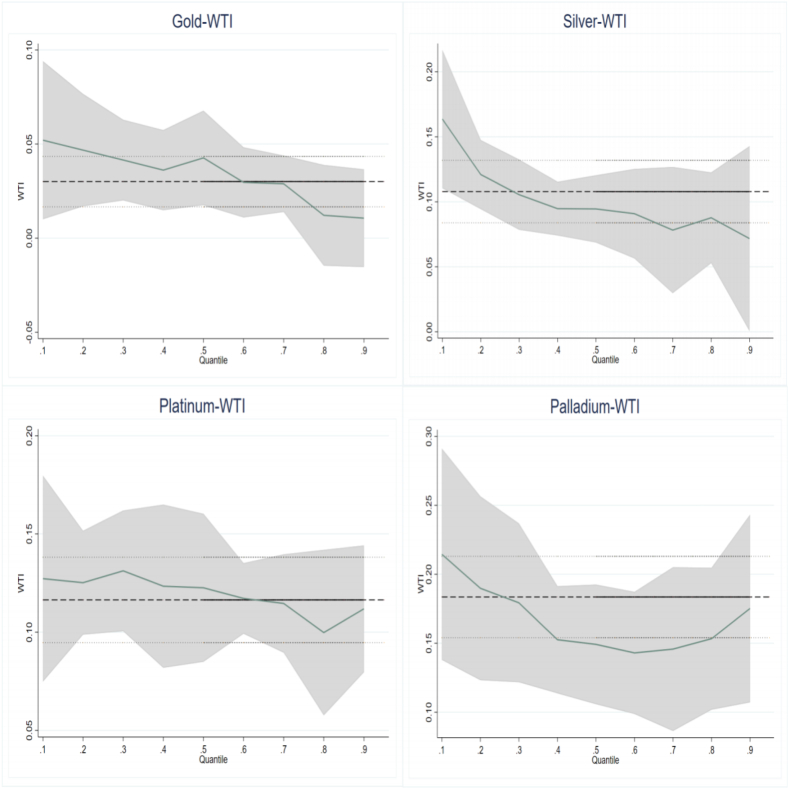
Quantile regression coefficient of WTI to precious metals for full sample.

Aimed at studying the impact of OP on PMR, this paper utilizes standard OLS and quantile regression (QR) models. The results are exhibited in Table 2 and Fig. 1.

**Table 2:** Results of oil price shocks to precious metals returns for the full sample.

Variables	OLS	0.1	0.2	0.3	0.4	0.5	0.6	0.7	0.8	0.9
Panel A: Gold										
WTI	0.030***	0.052***	0.047***	0.041***	0.036***	0.043***	0.030***	0.029***	0.012	0.011
	(4.401)	(4.085)	(5.098)	(5.394)	(5.216)	(6.261)	(4.526)	(4.492)	(1.377)	(0.815)
FFR	−0.003	−0.011	−0.003	0.000	−0.001	−0.004	−0.000	0.001	0.002	−0.003
	(-0.713)	(-1.341)	(-0.502)	(0.083)	(-0.229)	(-1.009)	(-0.072)	(0.133)	(0.318)	(-0.403)
VIX	0.001	−0.005***	−0.001	−0.001	−0.001	0.001	0.002***	0.003***	0.004***	0.006***
	(1.505)	(-4.062)	(-0.642)	(-1.204)	(-1.265)	(1.259)	(3.093)	(4.676)	(4.913)	(5.480)
Constant	−0.002	0.002	−0.005**	−0.001	0.001	−0.002	−0.003*	−0.003**	−0.004*	−0.007**
	(-1.447)	(0.535)	(-2.168)	(-0.738)	(0.301)	(-1.061)	(-1.702)	(-2.015)	(-1.930)	(-2.096)
Panel B: Silver										
WTI	0.108***	0.164***	0.121***	0.105***	0.095***	0.095***	0.091***	0.078***	0.088***	0.072***
	(8.798)	(7.802)	(8.406)	(7.772)	(8.505)	(9.149)	(8.710)	(5.977)	(6.137)	(2.973)
FFR	0.013*	−0.015	−0.024***	−0.020**	−0.014**	−0.006	0.008	0.009	0.001	0.034**
	(1.726)	(-1.130)	(-2.674)	(-2.373)	(-1.978)	(-0.889)	(1.183)	(1.084)	(0.076)	(2.322)
VIX	0.000	−0.010***	−0.006***	−0.005***	−0.003***	−0.000	0.002*	0.004***	0.008***	0.015***
	(0.061)	(-5.250)	(-4.427)	(-3.819)	(-2.625)	(-0.193)	(1.892)	(3.871)	(6.793)	(7.115)
Constant	−0.000	0.009*	0.005	0.006*	0.004	0.001	−0.002	−0.006*	−0.013***	−0.024***
	(-0.092)	(1.686)	(1.274)	(1.885)	(1.521)	(0.269)	(-0.648)	(-1.862)	(-3.592)	(-4.011)
Panel C: Platinum										
WTI	0.116***	0.127***	0.125***	0.131***	0.123***	0.123***	0.117***	0.115***	0.100***	0.112***
	(10.482)	(6.497)	(8.930)	(11.396)	(10.175)	(10.273)	(10.661)	(9.620)	(6.588)	(5.061)
FFR	0.005	−0.009	−0.024***	−0.018**	−0.012*	−0.018**	−0.005	−0.006	−0.002	−0.014
	(0.753)	(-0.729)	(-2.775)	(-2.537)	(-1.670)	(-2.483)	(-0.762)	(-0.817)	(-0.216)	(-1.003)
VIX	−0.001	−0.016***	−0.009***	−0.007***	−0.004***	−0.002*	0.001	0.004***	0.009***	0.016***
	(-1.451)	(-9.399)	(-7.425)	(-6.588)	(-4.201)	(-1.714)	(1.235)	(3.550)	(6.771)	(8.290)
Constant	0.004	0.027***	0.014***	0.012***	0.009***	0.005*	−0.000	−0.004	−0.014***	−0.027***
	(1.380)	(5.568)	(4.096)	(4.077)	(2.985)	(1.706)	(-0.013)	(-1.290)	(-3.828)	(-4.952)
Panel D: Palladium										
WTI	0.184***	0.215***	0.190***	0.179***	0.153***	0.149***	0.143***	0.146***	0.153***	0.175***
	(12.189)	(7.755)	(9.584)	(11.937)	(10.328)	(10.146)	(9.510)	(8.608)	(9.581)	(6.505)
FFR	0.006	0.006	−0.007	−0.013	−0.013	−0.004	0.012	0.006	0.010	0.006
	(0.609)	(0.341)	(-0.540)	(-1.420)	(-1.374)	(-0.484)	(1.270)	(0.594)	(0.974)	(0.356)
VIX	−0.004***	−0.023***	−0.015***	−0.011***	−0.008***	−0.004***	0.001	0.004***	0.008***	0.016***
	(-3.075)	(-9.442)	(-8.763)	(-8.752)	(-5.835)	(-3.270)	(0.781)	(2.705)	(5.500)	(6.706)
Constant	0.012***	0.041***	0.029***	0.024***	0.018***	0.012***	0.002	−0.002	−0.007*	−0.021***
	(3.144)	(6.070)	(5.890)	(6.512)	(4.812)	(3.404)	(0.556)	(-0.365)	(-1.690)	(-3.191)

Note: T-statistics in parentheses. ***p < 0.01, **p < 0.05, *p < 0.1, respectively.

From Table 2, OLS results revealed that OP positively affects PMR (i.e., the coefficient of WTI to Gold in Panel A is 0.030). OLS regression only reports the average effects [28]. While the estimated coefficients are statistically significant, the OLS method does not capture the relationship across diverse market states [53]. Table 2 reports the results of quantile regressions, which provide more details about the impact of OP in different precious metals market states. Consistent with Shahzad et al. [40] and Chen et al. [10], the positive influence of OP on the returns of the four precious metals is significant at least seven quantiles.

Another finding is that the response coefficients of precious metals in bear, normal, and bull market to OP are asymmetric, confirming the need for a QR approach. To identify the trend of the estimated coefficients concerning for the quantiles, Fig. 1 is plotted. Fig. 1 shows the quantile results for each region, where the horizontal axis represents the quantiles and the vertical axis denotes the response coefficient. The dashed line represents the OLS regression coefficient and the upper and lower limits of the 90 % confidence interval. Similarly, the curve represents the changes in the response coefficients, and the shaded part is the confidence interval of the quantile regression coefficient.

From Fig. 1, the regression coefficient gradually decreases as the quantiles increase. For example, the coefficient of WTI to Silver is 0.164 at the 0.1st quantile and decreases to 0.072 at the 0.9th quantile, suggesting that the strength of the effect of OP on PMR is most substantial in the case of bearish precious metals. Overall, Table 2 and Fig. 1 provide evidence of a positive asymmetric effect of OP on PMR. OP affects PMR mainly through inflation and economic growth. Inflation can be used to explain the positive effect of OP on PMR [7,26,27]. Rising crude oil prices push up energy costs, and inflation follows. Precious metals are relatively good hedges against inflation [27,30,32].

The oil price fluctuation is greater than that of precious metals [14,62]. Given the asymmetric nature of oil price shocks, this paper further analyses the impact of positive and negative oil price [45]. As Equation (8) describes, WTIZ represents positive oil price shocks, and WTIF represents adverse oil price shocks. Table 3 does not exhibit the results of control variables for saving space. From Table 3, results from OLS regression show positive and statistically significant coefficients of WTIZ and WTIF to gold, silver, platinum, and palladium. Further, at the quantile level, positive and negative OP shocks positively impact precious metal returns, which implies that OP may trigger PMR. As the financialization of commodities deepens and commodity markets become more closely linked, investors consider including both precious metals and oil in their portfolios, with the trend in precious metal prices gradually moving in line with oil prices [19,38]. OP is a mirror of the economy, and when OP rises due to global demand stimulus, the precious metals’ demand also increases [39], which in turn pulls up the returns of precious metals. Conversely, the positive impact of adverse OP shocks on PMR reflects the safe-haven nature of precious metals in the face of declining oil price shocks [14,63,64]. In other words, faced with ever-declining oil prices, investors choose precious metals as an alternative to oil.

**Table 3:** Asymmetric effects of oil price shocks to precious metals returns for full sample.

Variables	OLS	0.1	0.2	0.3	0.4	0.5	0.6	0.7	0.8	0.9
Panel A: Gold										
WTIZ	0.033***	0.010	0.020	0.031**	0.023**	0.043***	0.046***	0.044***	0.047***	0.070***
	(2.876)	(0.452)	(1.244)	(2.438)	(2.001)	(3.705)	(4.270)	(4.079)	(3.337)	(3.030)
WTIF	0.027**	0.104***	0.074***	0.058***	0.047***	0.037***	0.005	0.002	−0.035**	−0.027
	(2.270)	(4.785)	(4.539)	(4.475)	(3.997)	(3.092)	(0.490)	(0.146)	(-2.414)	(-1.140)
H1	0.12	7.26***	4.23**	1.66	1.58	0.12	5.33**	5.79**	12.54***	6.57**
H2	19.49***	27.18***	28.56***	36.50***	27.80***	33.90***	21.99***	18.93***	13.19***	9.22***
Panel B: Silver										
WTIZ	0.109***	0.148***	0.111***	0.103***	0.097***	0.093***	0.120***	0.123***	0.125***	0.108**
	(5.242)	(4.178)	(4.500)	(4.504)	(5.092)	(5.232)	(6.565)	(5.752)	(5.071)	(2.338)
WTIF	0.107***	0.203***	0.132***	0.114***	0.085***	0.096***	0.065***	0.046**	0.062**	0.063
	(5.047)	(5.657)	(5.284)	(4.911)	(4.425)	(5.329)	(3.481)	(2.111)	(2.490)	(1.337)
H1	0.01	0.91	0.28	0.09	0.14	0.01	3.45*	4.88**	2.43	0.36
H2	77.38***	71.51***	70.18***	64.84***	66.36***	81.52***	77.36***	50.21***	44.32***	10.25***
Panel C: Platinum										
WTIZ	0.091***	0.096***	0.089***	0.096***	0.099***	0.096***	0.107***	0.106***	0.170***	0.198***
	(4.841)	(2.597)	(3.853)	(4.764)	(4.952)	(4.927)	(5.697)	(5.110)	(6.919)	(5.489)
WTIF	0.142***	0.177***	0.169***	0.159***	0.156***	0.151***	0.119***	0.119***	0.065***	0.054
	(7.434)	(4.720)	(7.237)	(7.803)	(7.724)	(7.631)	(6.264)	(5.659)	(2.620)	(1.471)
H1	2.73*	1.80	4.56**	3.72*	3.12*	2.97*	0.16	0.15	6.80***	5.98**
H2	112.67***	40.85***	94.23***	118.91***	120.34***	118.03***	104.67***	84.87***	73.52***	41.53***
Panel D: Palladium										
WTIZ	0.144***	0.143***	0.108***	0.103***	0.117***	0.116***	0.118***	0.146***	0.175***	0.279***
	(5.622)	(3.150)	(3.426)	(3.802)	(4.634)	(4.785)	(4.641)	(4.838)	(6.773)	(6.530)
WTIF	0.224***	0.378***	0.324***	0.281***	0.186***	0.181***	0.171***	0.146***	0.116***	0.075*
	(8.656)	(8.221)	(10.117)	(10.274)	(7.268)	(7.352)	(6.652)	(4.784)	(4.433)	(1.726)
H1	3.74*	10.09***	17.51***	16.42***	2.81*	2.68	1.66	0.00	1.94	8.56***
H2	152.49***	104.27***	151.05***	160.72***	106.16***	110.15***	94.72***	67.67***	93.84***	58.59***

**Fig. 2:**
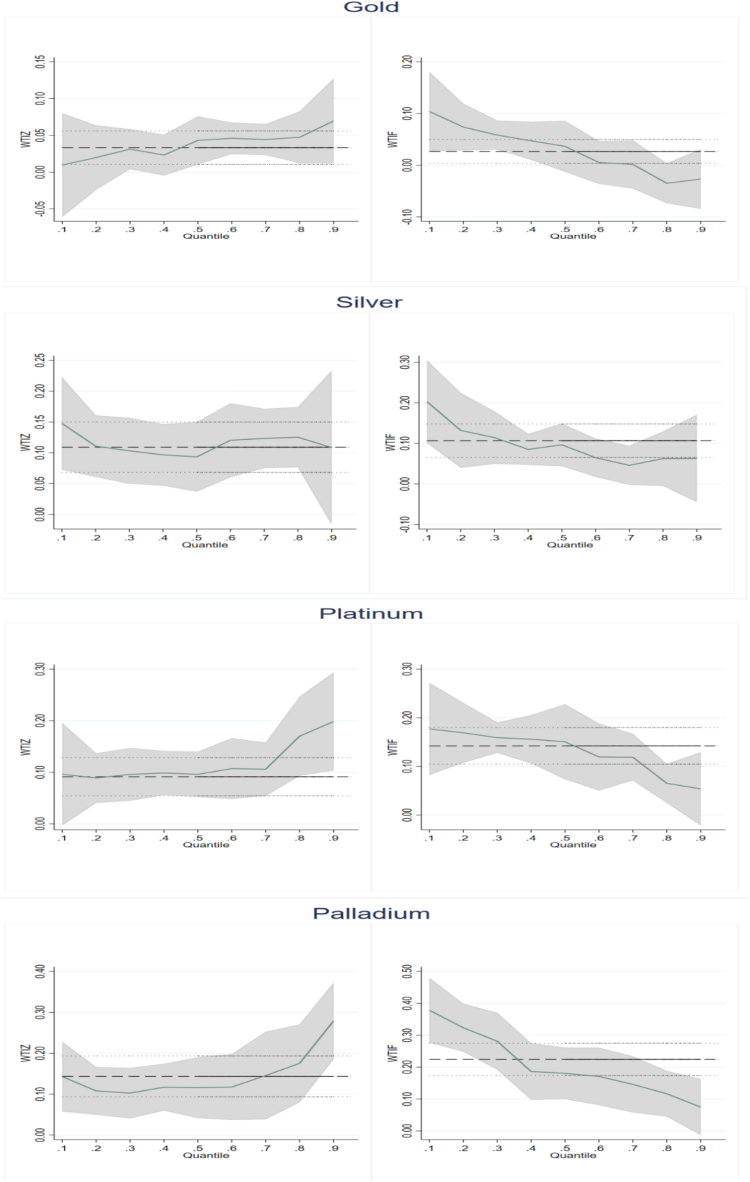
Quantile regression coefficient of positive and negative oil price shocks for full sample.

The results of decomposing OP on PMR at diverse quantiles are exhibited in Fig. 2. As shown in Fig. 2, the impact of positive OP shocks on PMR (except silver) rises with increasing quantiles, while the impact of adverse OP shocks on PMR falls with increasing quantiles. The asymmetric impact of oil price shocks on PMR is initially reflected.

In order to further determine the asymmetric OP effects, this paper uses the Wald test [10,45]. The results are shown in Table 3. The original hypothesis: H1: $\beta_{1\tau }$ = $\beta_{2\tau }$ rejection means that there is asymmetry at τ quantile. However, the non-rejection may be because $\beta_{1\tau }$ and $\beta_{2\tau }$ are both equal to zero. Therefore, we also carry out a more specific analysis of the situation where the coefficients are all equal to zero (H2: $\beta_{1\tau }$ = $\beta_{2\tau }$ = 0). The results show that H2 is rejected at all quantiles. As for H1, the significance for precious metals is proved at most quantiles. These findings further illustrate the significant asymmetric effect of oil price fluctuations.

### 4.2 Effects of USD exchange rate on precious metals for full sample

The impact of ER on PMR for the entire sample is reported in Table 4. From Table 4, the OLS results make it clear that the effect of ER on PMR is conspicuously negative, suggesting that an exchange rate rise would knock on precious metal returns. According to Zhu et al. [50], the OLS model might ignore the effects in heterogeneous distributions. This kind of defect can be avoided by applying quantile regression. The effect of ER is significantly negative across all quantiles. The negative coefficient clarifies that precious metals could be an alternative investment tool to pure dollar purchases [25]. This finding is consistent with Ciner et al. [3], Churchill et al. [7], and Hanif et al. [41]. Such a state of affairs could be due to the connection between the exchange rate and inflation. The inflation and exchange rate have strong mutual causality [28]; in this way, when inflation occurs, the dollar depreciates, and investors, fearing depreciation and protecting their purchasing power, turn to the precious metals markets, causing the price to rise.

**Table 4:** Results of the USD exchange rate to precious metals returns for the full sample.

Variables	OLS	0.1	0.2	0.3	0.4	0.5	0.6	0.7	0.8	0.9
Panel A: Gold										
ER	−0.751***	−0.799***	−0.884***	−0.891***	−0.884***	−0.867***	−0.848***	−0.760***	−0.710***	−0.684***
	(-15.692)	(-7.826)	(-14.672)	(-17.401)	(-20.069)	(-20.858)	(-18.183)	(-15.097)	(-10.835)	(-8.520)
FFR	−0.000	−0.008	−0.003	0.006	0.005	0.003	0.004	0.000	−0.003	−0.006
	(-0.070)	(-0.982)	(-0.600)	(1.338)	(1.320)	(0.724)	(1.142)	(0.008)	(-0.562)	(-0.844)
VIX	0.001**	−0.004***	−0.002**	−0.000	0.000	0.001	0.001**	0.003***	0.005***	0.006***
	(2.270)	(-3.693)	(-2.343)	(-0.475)	(0.833)	(1.392)	(2.338)	(5.188)	(6.026)	(6.472)
Constant	−0.003**	0.002	−0.001	−0.002	−0.003*	−0.002	−0.002	−0.005***	−0.007***	−0.007**
	(-2.154)	(0.682)	(-0.505)	(-1.419)	(-1.793)	(-1.205)	(-1.119)	(-2.799)	(-3.093)	(-2.469)
Panel B: Silver										
ER	−1.486***	−1.405***	−1.536***	−1.427***	−1.350***	−1.321***	−1.345***	−1.358***	−1.286***	−1.374***
	(-17.235)	(-8.932)	(-15.947)	(-15.651)	(-18.344)	(-17.185)	(-16.290)	(-16.188)	(-13.003)	(-8.499)
FFR	0.020***	−0.017	−0.014*	−0.015*	0.001	−0.005	−0.005	0.006	0.010	0.025*
	(2.702)	(-1.250)	(-1.683)	(-1.915)	(0.093)	(-0.822)	(-0.673)	(0.893)	(1.201)	(1.859)
VIX	0.001	−0.011***	−0.006***	−0.003***	−0.002**	0.000	0.003***	0.006***	0.009***	0.013***
	(0.544)	(-5.821)	(-4.882)	(-3.229)	(-1.967)	(0.211)	(2.701)	(5.632)	(7.409)	(7.001)
Constant	−0.002	0.013**	0.005*	0.004	0.002	−0.000	−0.004	−0.009***	−0.014***	−0.020***
	(-0.521)	(2.467)	(1.677)	(1.199)	(0.888)	(-0.155)	(-1.493)	(-3.215)	(-4.166)	(-3.666)
Panel C: Platinum										
ER	−1.498***	−1.287***	−1.339***	−1.379***	−1.471***	−1.426***	−1.340***	−1.356***	−1.368***	−1.392***
	(-19.308)	(-9.137)	(-12.081)	(-17.038)	(-17.918)	(-17.667)	(-15.490)	(-14.992)	(-15.006)	(-10.185)
FFR	0.012*	0.016	−0.001	−0.006	−0.006	−0.009	−0.014*	−0.006	−0.013*	−0.016
	(1.831)	(1.343)	(-0.082)	(-0.887)	(-0.803)	(-1.400)	(-1.882)	(-0.792)	(-1.682)	(-1.350)
VIX	−0.001	−0.015***	−0.009***	−0.006***	−0.004***	−0.001	0.001	0.005***	0.009***	0.014***
	(-1.083)	(-9.144)	(-7.142)	(-6.082)	(-4.344)	(-1.269)	(1.385)	(4.689)	(7.911)	(8.698)
Constant	0.003	0.026***	0.016***	0.010***	0.009***	0.003	−0.001	−0.007**	−0.013***	−0.022***
	(1.068)	(5.392)	(4.234)	(3.647)	(3.158)	(1.173)	(-0.253)	(-2.391)	(-4.373)	(-4.848)
Panel D: Palladium										
ER	−1.578***	−1.517***	−1.293***	−1.217***	−1.147***	−1.215***	−1.240***	−1.198***	−1.177***	−1.276***
	(-14.378)	(-7.630)	(-9.019)	(-10.582)	(-11.302)	(-10.874)	(-11.827)	(-10.891)	(-8.910)	(-6.448)
FFR	0.014	0.016	−0.001	0.006	0.018**	0.011	0.004	−0.005	−0.000	0.003
	(1.548)	(0.986)	(-0.114)	(0.591)	(2.100)	(1.135)	(0.429)	(-0.496)	(-0.005)	(0.172)
VIX	−0.004***	−0.022***	−0.014***	−0.009***	−0.007***	−0.004***	−0.001	0.003**	0.008***	0.014***
	(-3.115)	(-9.192)	(-8.399)	(-6.874)	(-6.222)	(-3.258)	(-0.651)	(2.373)	(5.265)	(5.844)
Constant	0.012***	0.039***	0.027***	0.019***	0.018***	0.013***	0.008**	0.001	−0.008*	−0.015**
	(3.221)	(5.798)	(5.563)	(4.853)	(5.180)	(3.463)	(2.137)	(0.188)	(-1.882)	(-2.325)

**Fig. 3:**
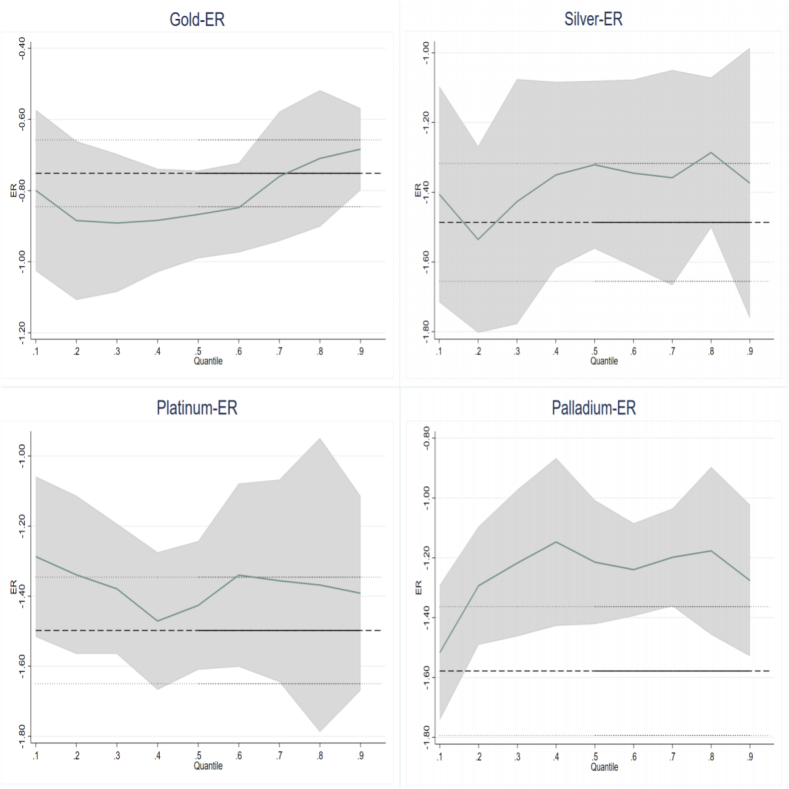
Quantile regression coefficient of ER to precious metals for full sample.

Additionally, there are apparent differences between different quantiles in the conditional distribution of PMR exhibited in Table 4. For example, in Panel C, platinum's most significant adverse reaction to exchange rate peaked at 0.4th quantile. It means platinum is the most highly hedged against the dollar in normal market conditions. Another finding is that the investors enjoy the safe heaven benefit of precious metals, mainly in bearish and normal conditions of precious metals. Fig. 3 presents the aggregated quantile regression coefficients. From this Figure, the coefficients of ER to Palladium gradually grow with the quantiles, indicating that the hedging power of palladium against the USD exchange rate decreases as the palladium market moves from a bearish to a bullish station. Overall, precious metals are a good hedge (based on the OLS condition mean and QR condition median) and a safe haven (based on 0.1st and 0.9th quantiles) in the case of dollar fluctuations [28].

### 4.3 Analysis of effects before and during COVID-19 epidemic

**Fig. 4:**
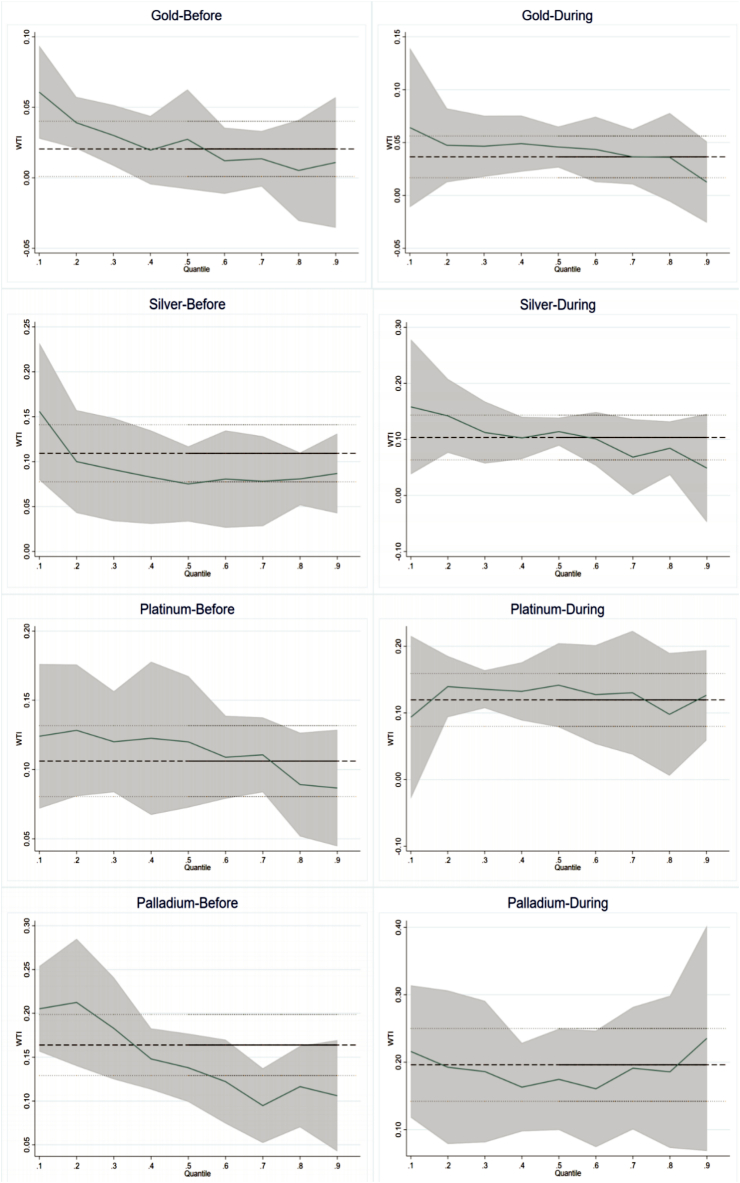
Quantile regression coefficient of WTI before and during COVID-19 period.

**Fig. 5:**
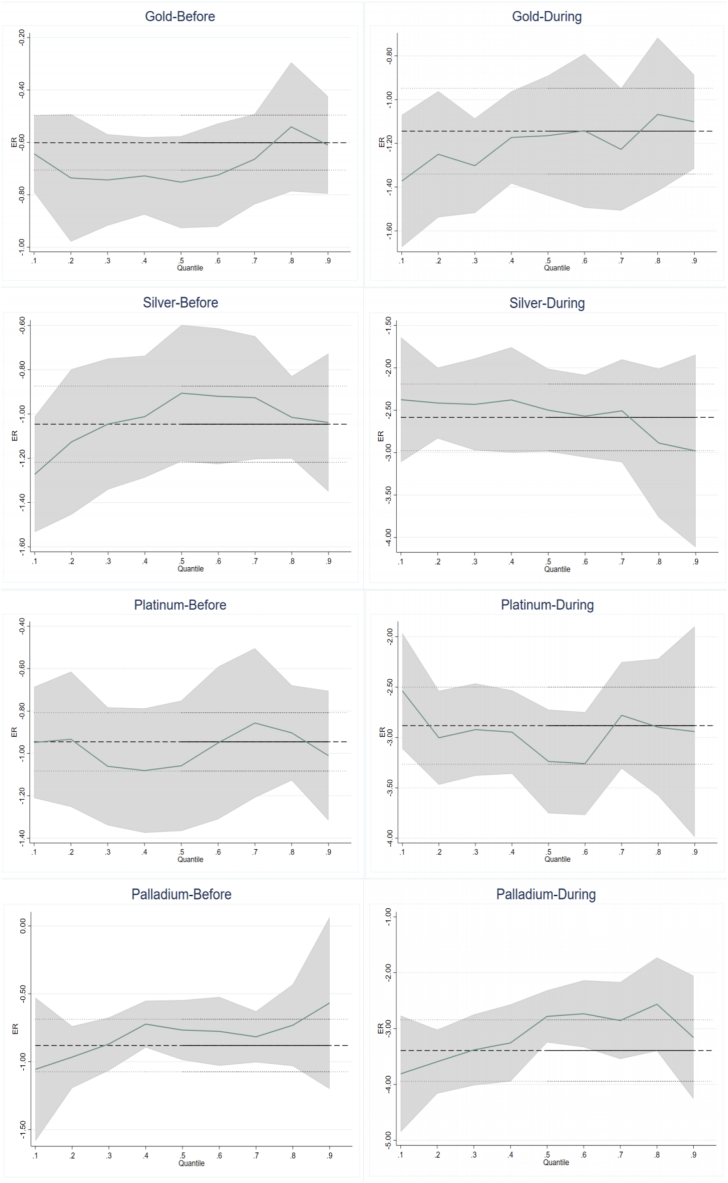
Quantile regression coefficient of ER before and during COVID-19 period.

The COVID-19 epidemic in early 2020 severely affected the world economy and destabilized financial markets [33]. Commodity prices are also volatile in this period [10]. To explore the impact of the pandemic, this paper conducts a sub-sample examination. World Health Organization (WHO) announced COVID-19 as a public health emergency of international concern on January 30, 2020 [65], and COVID-19 no longer constitutes a public health event of international concern on May 5, 2023. In this way, using January 30, 2020 as a watershed [66], this paper divides the total sample into two subsamples: the pre-COVID-19 period (January 1, 2013 to January 30, 2020) and the during-COVID-19 period (January 31, 2020 to May 5, 2023). The results are shown in Table 5, Table 6, Fig. 4, and Fig. 5.

**Table 5:** Results of oil price shocks to precious metals returns for sub-sample.

Variables	OLS	0.1	0.2	0.3	0.4	0.5	0.6	0.7	0.8	0.9
Panel A1: Gold-Before										
WTI	0.021**	0.061***	0.039***	0.030**	0.020**	0.027***	0.012	0.014	0.005	0.011
	(2.056)	(3.621)	(2.896)	(2.542)	(2.028)	(2.878)	(1.242)	(1.314)	(0.410)	(0.522)
Panel A2: Gold-During										
WTI	0.036***	0.064**	0.047***	0.047***	0.049***	0.046***	0.044***	0.037***	0.036***	0.013
	(3.633)	(2.567)	(3.328)	(4.386)	(4.570)	(4.321)	(4.704)	(4.095)	(2.739)	(0.615)
Panel B1: Silver-Before										
WTI	0.109***	0.156***	0.100***	0.091***	0.083***	0.075***	0.081***	0.078***	0.081***	0.087***
	(6.738)	(4.711)	(4.573)	(4.816)	(5.261)	(5.875)	(5.859)	(4.657)	(3.974)	(2.600)
Panel B2: Silver-During										
WTI	0.103***	0.158***	0.142***	0.112***	0.103***	0.114***	0.101***	0.068***	0.084***	0.049
	(5.084)	(4.568)	(5.756)	(5.504)	(5.280)	(5.970)	(5.213)	(2.929)	(3.156)	(1.403)
Panel C1: Platinum-Before										
WTI	0.106***	0.124***	0.128***	0.120***	0.123***	0.120***	0.109***	0.111***	0.089***	0.087***
	(8.123)	(4.986)	(7.301)	(7.650)	(7.071)	(7.961)	(7.753)	(7.830)	(5.438)	(3.219)
Panel C2: Platinum-During										
WTI	0.120***	0.094**	0.140***	0.136***	0.132***	0.142***	0.128***	0.130***	0.098***	0.127***
	(5.911)	(2.382)	(4.304)	(5.563)	(6.032)	(6.227)	(5.799)	(5.123)	(3.053)	(3.540)
Panel D1: Palladium-Before										
WTI	0.164***	0.205***	0.213***	0.183***	0.148***	0.138***	0.122***	0.095***	0.116***	0.106***
	(9.245)	(7.108)	(8.393)	(8.590)	(6.965)	(7.272)	(6.235)	(4.510)	(5.715)	(3.707)
Panel D2: Palladium-During										
WTI	0.196***	0.216***	0.192***	0.186***	0.163***	0.174***	0.160***	0.191***	0.186***	0.235***
	(7.127)	(4.403)	(4.782)	(6.644)	(6.634)	(6.399)	(5.319)	(6.842)	(5.154)	(5.263)

**Table 6:** Results of the USD exchange rate to precious metals returns for sub-sample.

Variables	OLS	0.1	0.2	0.3	0.4	0.5	0.6	0.7	0.8	0.9
Panel A1: Gold-Before										
ER	−0.601***	−0.644***	−0.736***	−0.744***	−0.728***	−0.752***	−0.725***	−0.665***	−0.541***	−0.610***
	(-11.232)	(-6.391)	(-10.178)	(-13.779)	(-14.268)	(-15.492)	(-13.528)	(-12.022)	(-7.365)	(-6.205)
Panel A2: Gold-During										
ER	−1.144***	−1.372***	−1.250***	−1.302***	−1.173***	−1.165***	−1.142***	−1.227***	−1.067***	−1.101***
	(-11.461)	(-6.205)	(-10.216)	(-11.397)	(-12.625)	(-13.243)	(-10.638)	(-10.639)	(-7.612)	(-6.406)
Panel B1: Silver-Before										
ER	−1.046***	−1.273***	−1.126***	−1.045***	−1.012***	−0.906***	−0.920***	−0.926***	−1.015***	−1.039***
	(-11.940)	(-7.173)	(-10.161)	(-11.141)	(-12.741)	(-11.165)	(-11.977)	(-10.842)	(-9.806)	(-6.121)
Panel B2: Silver-During										
ER	−2.584***	−2.376***	−2.415***	−2.431***	−2.378***	−2.499***	−2.570***	−2.507***	−2.887***	−2.980***
	(-12.897)	(-6.079)	(-10.173)	(-12.684)	(-12.380)	(-12.766)	(-12.783)	(-11.415)	(-10.863)	(-6.947)
Panel C1: Platinum-Before										
ER	−0.945***	−0.948***	−0.933***	−1.061***	−1.081***	−1.058***	−0.951***	−0.857***	−0.903***	−1.011***
	(-13.460)	(-6.932)	(-9.072)	(-12.909)	(-12.914)	(-13.483)	(-12.115)	(-10.023)	(-10.488)	(-6.174)
Panel C2: Platinum-During										
ER	−2.883***	−2.537***	−3.003***	−2.922***	−2.947***	−3.238***	−3.259***	−2.780***	−2.899***	−2.942***
	(-14.777)	(-7.611)	(-9.946)	(-11.637)	(-12.856)	(-14.437)	(-14.075)	(-11.365)	(-11.347)	(-7.913)
Panel D1: Palladium-Before										
ER	−0.881***	−1.057***	−0.966***	−0.870***	−0.723***	−0.767***	−0.777***	−0.817***	−0.731***	−0.567***
	(-8.924)	(-5.636)	(-7.521)	(-7.226)	(-6.835)	(-6.959)	(-7.437)	(-7.521)	(-6.150)	(-3.056)
Panel D2: Palladium-During										
ER	−3.393***	−3.810***	−3.593***	−3.382***	−3.256***	−2.782***	−2.735***	−2.856***	−2.565***	−3.160***
	(-12.106)	(-5.664)	(-9.332)	(-12.264)	(-12.033)	(-10.783)	(-9.797)	(-9.157)	(-7.009)	(-6.177)

From Table 5, Table 6, the impact of OP and ER on PMR is more substantial during COVID-19. For example, the OLS regression coefficients between precious metals and oil price shocks are significantly larger in COVID-19 than pre-COVID-19 (i.e., Panel A1 is 0.021 and A2 is 0.036 in Table 5). At the quantile level, this is more prevalent. Additionally, observing the coefficients of WTI and ER on PMR before and during the epidemic in Fig. 4, Fig. 5, it can be found that the epidemic has a heterogeneous impact on the relationship between these assets. In the case of platinum, for example, the coefficient WTI to platinum before the epidemic is most prominent at the 0.1st quantile and most minor at the 0.9th quantile. However, during the epidemic, this situation reverses. People think precious metals are durable communities that do not lose value [24,[67], [68], [69], [70]], and this is why investors often use precious metals as safe haven of financial assets. In this sense, a more significant correlation exists between PMR, OP, and ER in times of crisis. This paper also convinces it.

### 4.4 Robustness test

In order to verify robustness, based on Equation (6), this paper uses the daily price of Brent crude oil to replace WTI as the independent variable for regression again [10]. It is important to note that the Brent data sequence used here is treated like WTI described above. The quantile regression results are shown in Table 7.

**Table 7:** Robust.

Variables	OLS	0.1	0.2	0.3	0.4	0.5	0.6	0.7	0.8	0.9
Panel A: Gold										
BRENT	0.036***	0.072***	0.062***	0.044***	0.044***	0.042***	0.034***	0.034***	0.015	0.008
	(4.500)	(5.017)	(5.080)	(5.206)	(5.600)	(5.052)	(4.503)	(4.312)	(1.500)	(0.484)
Panel B: Silver										
BRENT	0.119***	0.179***	0.127***	0.113***	0.102***	0.099***	0.100***	0.085***	0.109***	0.089***
	(8.147)	(7.814)	(6.780)	(7.263)	(7.694)	(7.870)	(7.557)	(5.600)	(6.724)	(3.261)
Panel C: Platinum										
BRENT	0.145***	0.150***	0.139***	0.144***	0.128***	0.133***	0.142***	0.130***	0.117***	0.128***
	(11.048)	(6.707)	(7.504)	(9.768)	(9.239)	(9.882)	(10.721)	(9.184)	(6.564)	(5.024)
Panel D: Palladium										
BRENT	0.219***	0.232***	0.223***	0.203***	0.175***	0.178***	0.162***	0.153***	0.147***	0.187***
	(12.189)	(7.300)	(9.174)	(11.734)	(10.398)	(9.943)	(8.974)	(7.243)	(7.632)	(5.440)

Table 7 explicitly shows that oil price's impact on precious metals is significantly positive. Such regression results are consistent with the results in the first section of this chapter, which indicates that the empirical results of this paper are robust.

## 5 Conclusions and implications

Using the quantile regression method, this paper studies the asymmetric impact of oil prices (OP) and USD exchange rate (ER) on precious metals returns (PMR) from January 1, 2013 to May 5, 2023. Moreover, we compare the difference in the impact during the COVID-19 epidemic by examining two sub-samples. The conclusions are as follows.

First, oil prices positively affect precious metals returns, proving that higher oil prices lead to higher precious metal returns. In particular, the effect is most potent when precious metals are in extreme market conditions, such as a bear market. Second, the effects of positive and negative oil price shocks on precious metals are asymmetric. Specifically, the contribution of favorable oil prices to the returns of the primary precious metals increases with the number of quantiles, and the positive impact of adverse oil price shocks on precious metals decreases with the number of quantiles. Third, the effect of the USD exchange rate on precious metals is negative and is most vital when precious metals are in bear and normal conditions, suggesting that precious metals can act as a currency safe haven and a hedge against inflation risk. Finally, by further comparing and analyzing the effects on the returns of precious metals before and during COVID-19, this paper finds that the effects of oil price shocks and exchange rate exhibit different characteristics over time. The results of the sub-sample regressions suggest that COVID-19 significantly enhances the impact of oil prices and exchange rate on precious metals, but does not change the direction of the effect.

The implications can be summarized as follows. First, oil price shocks and exchange rate are essential factors affecting the precious metals market, so investors and producers should consider them when avoiding risks. Investors and producers are advised to closely monitor changes in oil prices and respond by rebalancing their portfolios to adjust precious metal allocations based on expected inflationary effects. Second, the impact of market conditions should also be considered. Due to the asymmetry and heterogeneity of oil prices and exchange rate on precious metals under different market conditions, policymakers should formulate corresponding plans suiting to different market conditions. Finally, a black swan event such as COVID-19 can create huge systemic risks and impact commodities as well as financial markets. Therefore, investors and policymakers are advised to pay more attention to the events and set up optional investment portfolios to deal with such contingencies.

It has to be admitted that there are some limitations to this article. For example, the data in this article uses precious metal futures data and ignores physical commodities. Precious metals futures have financial attributes, while physical precious metals have vital commodity attributes. Differences in trading characters may affect their pricing and the interpretation of the empirical results. Therefore, future research could be less limited to precious metal futures and more inclusive of physical objects in the research framework. In addition, the trend towards the financialization of commodities has increased the price volatility of precious metals, and it is uncertain whether new macroeconomic factors will affect their prices. Future research could incorporate other economic factors to predict changes in precious metal prices.

## Declaration of competing interest

The authors declare that they have no known competing financial interests or personal relationships that could have appeared to influence the work reported in this paper.
